# A Narrative Review of the Diagnosis and Treatment of Sarcopenia and Malnutrition in Patients with Heart Failure

**DOI:** 10.3390/nu16162717

**Published:** 2024-08-15

**Authors:** Lucía de Jorge-Huerta, Cristian Marco-Alacid, Cristina Grande, Christian Velardo Andrés

**Affiliations:** 1 Hospital Universitario 12 de Octubre, 28041 Madrid, Spain; 2 Hospital Virgen de los Lirios, 03804 Alcoy, Spain; marco_criala@gva.es; 3 Medical Scientific Liaison, Abbott Nutrición, 28050 Madrid, Spain; cristina.grande@abbott.com; 4 Hospital Virgen del Puerto, 10600 Plasencia, Spain; christian.velardo@salud-juntaex.es

**Keywords:** sarcopenia, heart failure, malnutrition, physical exercise, diet, nutritional supplementation

## Abstract

The prevalence of sarcopenia (loss of muscle strength, mass and function) in individuals with heart failure (HF) stands at a considerable level (approximately 20%), contributing to heightened mortality rates and diminished quality of life. The underlying pathophysiological mechanisms involve the presence of low-grade inflammation and a disturbance of the anabolic–catabolic protein balance. The nutritional assessment of patients with HF is a key aspect, and diverse diagnostic tools are employed based on patient profiles (outpatient, inpatient and nursing home). The Global Leadership Initiative on Malnutrition (GLIM) criteria serves as a consensus for diagnosing malnutrition. Given that edema can impact body mass index (BMI) in patients with HF, alternative body assessment technical methods, such as bioelectrical vector impedance (BiVA), BIA (without vector mode), computer tomography (CT) or clinical ultrasound (US), are useful. Scientific evidence supports the efficacy of both aerobic and resistance physical exercises in ameliorating and preventing muscle wasting associated with HF. Dietary strategies emphasize the importance of protein intake, while certain micronutrients like coenzyme Q10 or intravenous iron may offer benefits. This narrative review aims to present the current understanding of the pathogenesis, diagnosis and treatment of muscle loss in individuals with heart failure and its consequential impact on prognosis.

## 1. Introduction

According to the universal definition and classification of heart failure (HF), it is not simply a single pathological diagnosis; rather, it is a clinical syndrome that includes symptoms and/or signs such as breathlessness, ankle swelling and fatigue, which may be accompanied by signs such as elevated jugular venous pressure, peripheral edema and pulmonary crackles. It is caused by a structural and/or functional abnormality of the heart that leads to elevated intracardiac pressures and/or inadequate cardiac output, whether at rest, during exercise or in both cases. This definition is supported by increased levels of natriuretic peptide levels and/or objective signs of pulmonary or systemic congestion [1].

Classifications of the disease based on left ventricular ejection fraction (LVEF) are as follows:

- HFrEF (heart failure with reduced ejection fraction): Symptomatic HF with LVEF ≤ 40%.
- HFmrEF (heart failure with mildly reduced ejection fraction): Symptomatic HF with LVEF 41–49%.
- HFpEF (heart failure with preserved ejection fraction): Symptomatic HF with LVEF ≥ 50%.

With a significant impact on public health, HF affects up to 2% of the general population and a staggering 10% of individuals aged 70 and above, making it a leading cause of hospitalizations and deaths in industrialized countries [1]. However, the intricate interplay between HF and associated conditions, such as sarcopenia, sarcopenic obesity and cachexia, introduces further complexity. According to the definition of the European Working Group of Sarcopenia in Older People (EWGSOP2), sarcopenia is considered a progressive and generalized skeletal muscle disorder characterized by the gradual loss of muscle strength, mass and function. It is intricately linked to adverse outcomes, including falls, fractures, physical disability and heightened mortality [2].

**Figure 1:**
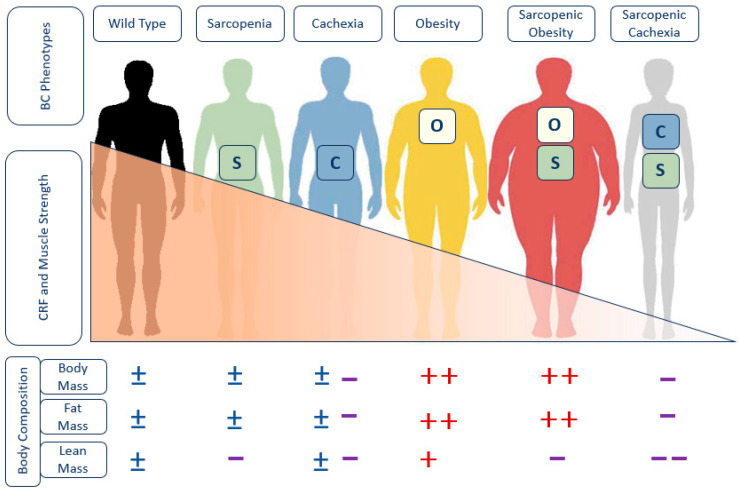
Body composition phenotypes and the progressive loss of cardiorespiratory fitness and muscle strength in heart failure. BC: Body Composition; O: Obesity; C: Cachexia; S: Sarcopenia; CRF: Cardiorespiratory Fitness; +: Increase; −: Decrease; ±: Unchanged.

Clinically, cachexia is defined as a complex metabolic syndrome primarily caused by exacerbated catabolism, leading to involuntary weight loss (potentially accompanied by muscle loss) that exceeds 5% of the usual weight over the previous year. Additionally, for diagnosis, at least three of the following symptoms must be present: anorexia, fatigue, reduced muscle strength, loss of fat-free mass and biochemical alterations in inflammatory markers (IL-6, CRP), hemoglobin or serum albumin [3]. The syndrome presents with mortality rates that are 2–3 times higher compared to patients with non-cachectic HF. The confluence of low muscle mass and high body fat gives rise to the phenomenon of sarcopenic obesity (SO) [4,5,6] (Figure 1).

While there is some definitional overlap, it is crucial to recognize sarcopenia and cachexia as distinct clinical entities. Unlike cachexia, weight loss is not a prerequisite for diagnosing sarcopenia, emphasizing the importance of precise clinical distinctions.

Adding another layer of complexity, malnutrition emerges as a significant factor impacting muscle assessments in HF patients. Malnutrition can arise from insufficient nutrient intake or poor assimilation, and various disease-associated mechanisms, including inflammation, can further complicate this condition. Malnutrition is linked to negative functional and clinical outcomes, especially when it accompanies illnesses or injuries. In such cases, malnutrition is characterized by a combination of reduced food intake/assimilation with varying degrees of inflammation, either acute or chronic. This complex interplay results in altered body composition and decreases biological function, as evidenced by decreased muscle mass markers such as fat-free mass (FFM), muscle mass index (MMI) or body cell mass (BCM) [7].

The objective of this narrative review is to provide an updated synthesis of evidence about the epidemiology, pathophysiology, diagnosis and treatment of sarcopenia and malnutrition in patients with HF. By exploring the intricate relationships between HF, sarcopenia, cachexia, and malnutrition, this review aims to contribute to a comprehensive understanding of these conditions, guiding future research directions and clinical interventions in this vital area of cardiovascular medicine.

## 2. Methodology

We conducted a search on PubMed, Medline, and Cochrane Plus databases for original articles, reviews and meta-analyses by combining descriptors that corresponded to the controlled language thesaurus of the Medical Subjects Headings (MeSHs) with keywords such as prevalence, heart failure, body mass index, weight loss, muscle strength, skeletal muscle, sarcopenia, muscle wasting, malnutrition, physical exercise, diet and nutritional supplementation. The Boolean operators “AND” and “OR” were used: ((diagnosis[Title/Abstract]) OR (treatment[Title/Abstract]) OR (sarcopenia[Title/Abstract]) OR (malnutrition[Title/Abstract])OR (heart failure[Title/Abstract])) AND ((body mass index[MeSH Terms]) OR (agents, weight loss[MeSH Terms]) OR (muscle strength[MeSH Terms]) OR (atrophic muscular disorder[MeSH Terms]) OR (body weight change[MeSH Terms]) OR (muscle weakness[MeSH Terms] OR (cardiac amyloidosis[MeSH Terms]) OR (sarcopenia[MeSH Terms]) OR (muscle wasting[MeSH Terms]) OR (malnutrition [MeSH Terms]) OR (physical exercise [MeSH Terms]) OR (nutritional supplementation [MeSH Terms]).

**Figure 2:**
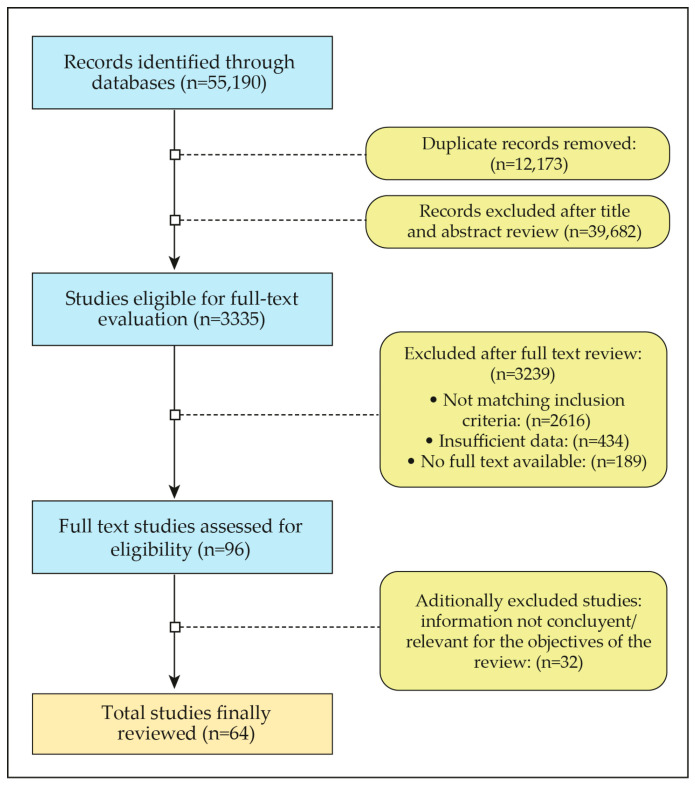
Eligibility criteria flowchart.

The search was carried out between 2001 and 2024. In total, 55,910 articles were initially identified, followed by the exclusion of references that did not fit the inclusion criteria and those in which the full text was not available. Of the total number of papers identified and after the exclusions resulting from the review of the pool of authors, a total of 64 articles that reviewed key aspects were finally selected for the narrative review (Figure 2).

## 3. Understanding the Disease (Epidemiology, Pathophysiology and Prognosis)

### 3.1. Epidemiology

Sarcopenia impacts over 20% of individuals with heart failure (HF) and is a significant predictor of decreased physical capacity and increased mortality among HF patients. Comparable prevalence rates of around 20% have been documented across patients with HF, regardless of whether they exhibit heart failure with a reduced ejection fraction (HFrEF) or heart failure with a preserved ejection fraction (HFpEF). The prevalence of cachexia among patients with HF is estimated to be between 5 and 15%. The coexistence of cachexia/sarcopenia is commonly observed, particularly among elderly patients with cardiovascular diseases [4,5].

Turning our attention to the nutritional landscape, severe malnutrition is estimated to afflict approximately 7.5% of HF patients, while moderate malnutrition has a notably higher incidence of 57% [8]. Importantly, these nutritional challenges may or may not be intricately linked with the presence of sarcopenia [4,5].

### 3.2. Pathophysiology: The Cardiac-Skeletal Muscle Binomial

**Figure 3:**
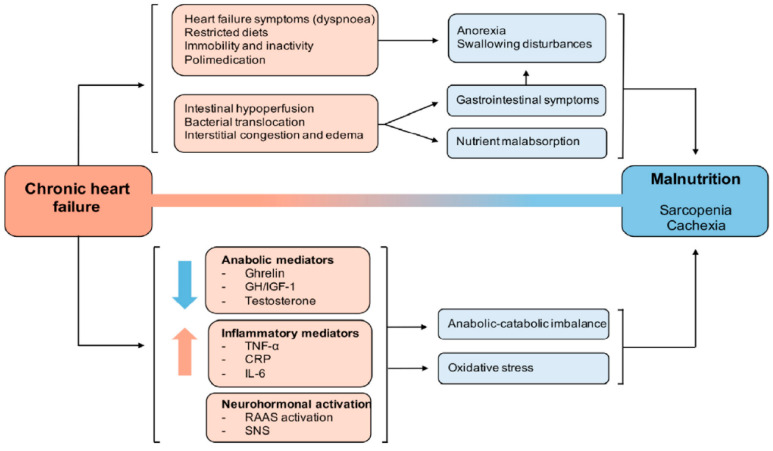
Main pathophysiological alterations that occur in the development of malnutrition in relation to heart failure [8]. GH: growth hormone; IGF-1: insulin-like growth factor-1; TNF-α: tumor necrosis factor alpha; CRP: C-reactive protein; IL-6: interleukin-6; RAAS: renin–angiotensin–aldosterone system; SNS: sympathetic nervous system. Reproduced with permission from Fernández Pombo A et al. Relevance of nutritional assessment and treatment to counteract cardiac cachexia and sarcopenia in chronic heart failure. Clin. Nutr. 2021, 40, 5141–5155. CC-BY-NC-ND License.

Various biological processes of HF are early inductors of sarcopenia (Figure 3), contributing to heightened patient mortality. Inadequate protein intake due to poor appetite induced by diminished cardiac output or due to dyspnea during intake, coupled with nutrient malabsorption due to factors like intestinal edema, prolonged pro-inflammatory states, physical inactivity and potential comorbidities may eventually lead to cachexia and sarcopenia in patients with HF [9].

In addition, cardiac dysfunction in HF not only impacts the minimum cardio-respiratory fitness level necessary for exercise but also instigates physical inactivity and prolonged bedrest, thereby fostering muscle wasting and dysfunction [9].

Endothelial dysfunction emerges as a potential early marker of sarcopenia, as reduced cardiac output and endothelial dysfunction in HF may impede skeletal muscle blood flow. This diminished blood flow, in turn, hinders oxygen delivery to the muscles, leading to muscle tissue ischemia [9].

Glucose intolerance and hyperglycemia surface are risk factors not only for acceleration of skeletal muscle loss and progressive weakness but also for impaired physical function, as evidenced by slower walking speeds [10].

Chronic smoking has also been reported to induce oxidative damage to skeletal muscle proteins, contributing to muscle mass loss and dysfunction and the development of sarcopenia in the general population but also in pathological populations like HF patients or those with chronic obstructive pulmonary disease (COPD) [11].

The increased catabolic stress in the skeletal muscle of HF patients results in exercise intolerance, ventilatory inefficiency and chronotropic incompetence, as well as insulin resistance, suggesting a significant contribution of the catabolic status mechanism to the limited functional status of patients [5].

The presence of chronic low-grade inflammation emerges as a common feature in the progression of sarcopenia. The aging process is hypothesized to be associated with chronic low-grade inflammation, known as inflammaging, with epidemiological studies linking inflammaging to various health risks, including cardiovascular disease (CVD), chronic kidney disease, cancer, depression, dementia, sarcopenia, mobility disability and frailty. Consequently, CVD and sarcopenia are posited as diseases induced by inflammaging, synergistically contributing to adverse health outcomes [11].

The overproduction of age-related reactive oxygen species (ROS) in skeletal muscle leads to oxidative damage, contributing to muscle atrophy, motoneuronal degeneration and impaired muscle contractility. Patients with heart failure show increased serum levels of oxidative stress markers, including plasma malondialdehyde and erythrocyte superoxide dismutase activity. Elevation of serum oxidative stress markers was correlated with reduced exercise tolerance in these patients [11].

It is known that skeletal muscle releases myokines, which are biologically active signaling molecules and peptides with paracrine and endocrine activity involved in the metabolic homeostasis of other tissues in the body [12,13].

Several cells and tissues are capable of releasing myokines; however, given that skeletal muscle comprises approximately 40% of the total body mass, it is likely the largest producer of myokines in the human body [14].

Each type of muscle fiber is capable of secreting different types of myokines [15]. In relation to heart disease, the myokines primarily produced by glycolytic fibers are osteoprotegerin, musclin and angiogenin, whereas oxidative fibers mainly produce irisin and myonectin.

Physical activity and muscle disuse result in various adaptations by either enhancing or reducing the production of myokines [14].

In heart failure, myopathy is considered a secondary muscle injury associated with low capillary perfusion. It is characterized by reduced muscle strength, atrophy of type 1 and 2a fibers, microvascular inflammation, increased oxidative stress and a decreased capacity for repair following muscle damage. However, in the early stages of the disease, myokines are capable of ensuring that both the structure and function of skeletal muscles undergo adaptive metabolic regulation. As the disease progresses, the myokine profile may become altered, and this alteration is associated with the onset of sarcopenia and cachexia in these patients [16].

The alteration in myokine expression will result in the dysregulation of protein homeostasis in both skeletal muscle and the myocardium, further exacerbating cardiac dysfunction, muscle weakness and physical activity intolerance in cardiac patients.

The known myokines related to heart failure originating from skeletal muscle are primarily decorin, irisin, myonectin, brain-derived neurotrophic factor (BDNF), growth differentiation factor-11 (GDF-11), myostatin, and osteonectin (Table 1).

Other myokines whose activity is related to heart failure but originate from tissues other than muscle include the following: interleukins 8 and 15 (IL-8, IL-15) and fibroblast growth factor 21 (FGF-21) (Table 2).

A range of other biochemical parameters, including ghrelin, leptin, adiponectin, interleukin-1 (IL-1), interleukin-6 (IL-6), C-reactive protein (CRP), tumor necrosis factor-alpha (TNF-α), insulin growth factor type 1 (IGF-1) and testosterone have also been studied as potential biomarkers of nutritional status in HF patients [8,17].

Biomarkers IL-1, IL-6, CRP and TNF-a exhibit notable elevation in HF patients, particularly in those presenting with cachexia and sarcopenia [8].

Increased levels of these parameters correlate with low handgrip strength in people over the age of 65 and with higher mortality in HF patients [6,8,9]. Significantly, sarcopenia in heart failure patients with coronary artery disease (CAD) and unstable atherosclerotic plaques is marked by decreased IGF-1 expression in skeletal muscle, suggesting a potential role of IGF-1 as a positive modulator of muscle strength and function [8].

### 3.3. Prognosis

The contribution of sarcopenia to increased mortality in patients with HFpEF and HFrEF is similar [18].

The loss of muscle mass demonstrates a more profound reduction in functional capacity, as assessed by parameters such as handgrip strength, quadriceps strength, or a 6 min walk test. In addition, health-related quality of life (QoL), as assessed using the EQ-5D questionnaire, is significantly reduced in those cases where muscle loss is present, in comparison to weight loss alone [19]. Furthermore, muscle loss serves as a predictor of hospital readmission and long-term mortality, particularly among elderly patients in acute care wards [5].

Likewise, the presence of malnutrition is linked to a mortality increase ranging from 2 to 10 times higher than that seen in HF patients without malnutrition [20].

Paradoxically, despite the heightened risk of heart failure associated with obesity, mortality rates in obese HFrEF patients may be lower than in those with a normal healthy body mass index (BMI) (18.5–24.99 kg/m^2^) [18,21]. Nevertheless, the protective effect of a higher BMI disappears in the presence of a low lean mass and a high fat mass [22]. Notably, the combination of sarcopenia and obesity identifies a subgroup at higher risk, as the independent adverse effects of these two conditions may intestinally potentiate each other [6,22].

Therefore, early diagnosis and prevention of sarcopenia and malnutrition are needed for patients with CVD to prevent a worsening prognosis. In non-obese men, sarcopenia has been linked as a potential risk factor for cardiovascular disease [23]; it also may have predictive value for adverse outcomes in heart failure [5].

As a reminder, the prevalence of sarcopenia is high in both HF patients with left ventricular ejection fraction (LVEF), reduced fraction (HFrEF), and those with a preserved fraction (HFpEF) (approximately 20%), leading to increased mortality and decreased quality of life.

## 4. Diagnostic Methods

### 4.1. Nutritional Assessment: Global Leadership Initiative on Malnutrition (GLIM) Criteria

Given the intricate interrelation between nutritional status and muscle mass, a comprehensive assessment of nutritional status is imperative in the early stages of HF and throughout the follow-up period.

To effectively diagnose malnutrition and sarcopenia, it is crucial to emphasize the consolidation and classification of diagnostic criteria, given the numerous tools available for identifying malnutrition in routine clinical practice [4].

**Figure 4:**
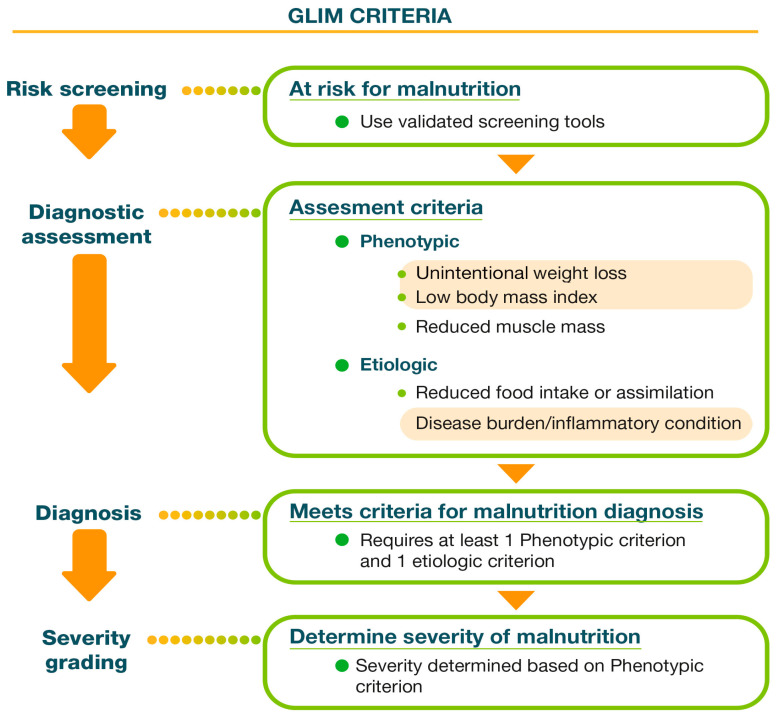
Diagnostic flowchart of GLIM criteria and phenotypic and etiologic criteria.

The GLIM criteria, started in 2016 through a collaborative effort by the four most important international clinical nutrition societies: European Society of Parenteral and Enteral Nutrition (ESPEN), American Society of Parenteral and Enteral Nutrition (ASPEN), Federación Latinoamericana de Terapia Nutricional, Nutrición Clínica y Metabolismo (FELANPE) and Parenteral and Enteral Nutrition Society of Asia (PENSA), were established to create a consensus on the screening and diagnosis of malnutrition. Importantly, these criteria apply to HF patients [7] (Figure 4).

#### 4.1.1. Screening

Various tools for malnutrition assessments are widely used in clinical screening, with consideration for the specific setting in which each tool is administered (inpatient, outpatient or nursing home).

These tools include the Short Nutritional Assessment Questionnaire (SNAQ), the Malnutrition Universal Screening Tool (MUST), the Mini Nutritional Assessment (MNA), the Nutritional Risk Screening 2002 (NRS 2002), the Nutrition Risk in the Critically Ill (NUTRIC) score and the Subjective Global Assessment (SGA). Although not specifically validated for HF, the Mini Nutritional Assessment-Short Form (MNA-SF) is an independent predictor of mortality in HF patients with preserved left ventricular ejection fraction (LVEF; HFpEF) [1,4,8].

Assessment of the nutritional status in obese patients is equally important as a substantial proportion of HF patients with obesity are malnourished or at risk of malnutrition, with estimates ranging from 10% to 50%, depending on the population and screening instrument used [24].

#### 4.1.2. Diagnosis

For the diagnosis of malnutrition according to GLIM criteria, the presence of at least one phenotypic criterion (low body mass index (BMI), unintentional weight loss and reduced muscle mass) and one etiologic criterion (reduced food intake or absorption and presence of inflammatory burden) is required.

The severity of malnutrition is determined by the following phenotypic criteria:

Moderate malnutrition:

Weight loss of 5–10% in the last 6 months or 10–20% over more than 6 months.

BMI < 20 for those under 70 years old or <22 for those older than 70 years.

Mild to moderate muscle mass loss.

Severe malnutrition:

Weight loss of ≥10% in the last 6 months or ≥20% over more than 6 months.

BMI < 18.5 for those under 70 years old or <20 for those older than 70 years.

Severe muscle mass loss.

##### Phenotypic Criteria, a Challenge in Patients with HF

While monitoring the weight of HF patients annually is reasonable, assessing malnutrition in this population is complicated due to the potential masking effect of edema on the underlying loss of muscle mass and weight. Routine clinical assessments of patient weight and body mass index (BMI) may remain stable despite malnutrition due to the state of congestion. This issue extends to the estimation of muscle mass using calf circumference, as lower limb edema in HF patients may falsely present muscle reserve within the normal range [22].

**Figure 5:**
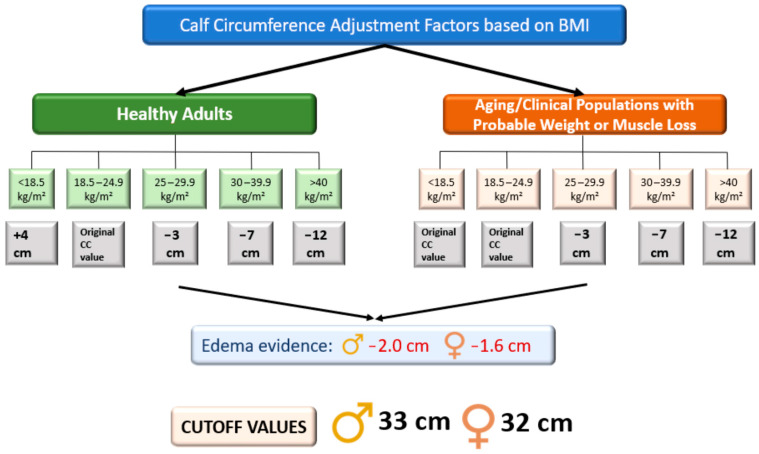
Calf circumference adjustment factors based on BMI [25,26,27].

It is important to consider the adjustment of calf circumference in patients with edema (−2 cm in men, −1.6 cm in women) [25] and/or overweight or obesity (BMI 25–29.9 kg/m^2^: −3 cm; BMI 30–39.9 kg/m^2^: −7 cm; BMI >40 kg/m^2^: −12 cm) (Figure 5). This is particularly important to avoid erroneous measurements in patients with HF and/or sarcopenic obesity [26].

To address this challenge, several methods have been developed for indirect body composition assessment in HF patients, including bioelectrical impedance (BIA), dual-energy X-ray absorptiometry (DXA), computed tomography (CT), magnetic resonance imaging (MRI) and muscle ultrasound:

- BIA, a fast, safe, affordable and easy-to-use option, estimates body composition based on mathematical calculations. While there are publications indicating that BIA is safe for patients with cardiac implants [28], some caution is still warranted. BIA measurements before and after cardiac device implantation reveal significant differences in terms of resistance and reactance values, suggesting changes in total body water (TBW), extracellular fluid (ECF), FFM, and fat mass (FM). However, the impact of hydration status changes on these results cannot be ruled out [29]. Manufacturers remain cautious about BIA measurements in patients with implanted cardiac devices due to potential interference from metal implants. Therefore, interpreting BIA results in this patient group should be performed carefully, considering the presence of large metal implants that may affect the flow of electrical current [30]. However, it requires validation in the specific population being studied and may be of limited use in patients with hydration disorders [5]. In HF patients, BIA body composition (BC) data provide information about the different body compartments (FFM and FM) and about hydration: TBW, ECW and ICW. No clinically evident edema, identified through BIA as an elevated ratio of ECW to TBW, may serve as an early indicator of preclinical cardiovascular (CV) disease. Additionally, this condition is associated with increased CV mortality. Notably, higher TBW has been observed in patients with atrial fibrillation. Furthermore, assessing the proportions between TBW compartments in CV patients reveals that left ventricle ejection fraction correlates positively with ICW and negatively with ECW in individuals with coronary artery disease [30]. The ratio of ECW to TBW could be obtained from BIA, and an edema index of >0.39 is clinically recognized as the threshold for fluid overload [31].
  We can also obtain direct muscle data from parameters such as the Appendicular Skeletal Muscle Index (ASMI), Fat-Free Mass Index (FFMI) and Appendicular Lean Mass (ALM). All of these parameters have been of high value because they focus on the distribution of body compartments, which are of great relevance in nutritional assessments in the GLIM criteria. In cases of overhydration, raw impedance parameters, such as resistance (Rz), reactance (Xc) and the phase angle (PhA), can be obtained using the following:
  PhA = arc tangent (Xc/R) × 180°/π.
  PhA is positively associated with tissue reactance (associated with cell mass, integrity, function and composition) and negatively associated with resistance, which depends mainly on the degree of tissue hydration [32]. PhA serves as a valuable prognostic factor. Research has demonstrated a strong correlation between low PhA values and increased CV risk [33]. Among patients with chronic HF, lower PhA levels are evident, particularly in those indicated as having a higher New York Heart Association (NYHA) class. Consequently, PhA can be utilized when assessing the severity of chronic HF. Furthermore, higher PhA values are associated with improved prognosis in chronic HF cases. Notably, during acute HF episodes, PhA values tend to be lower compared to chronic HF, and reduced PhA is linked to higher mortality during acute decompensated HF [30]. A derivative of BIA, bioelectrical impedance vector analysis (BIVA), can be used as an alternative [24]. It seems to provide more accurate values in terms of screening, managing and monitoring HF. BIVA and hydrograph techniques hold promise in the management of heart failure. BIVA aids in identifying both chronic and acute heart failure, while hydrograph facilitates treatment adjustments by monitoring fluid status. The American Heart Association recommends the use of BIVA to optimize fluid therapy and prevent cardiorenal syndrome. Combining BIVA with serum B-type natriuretic peptide (BNP) or N-terminal pro-BNP (NT-proBNP) levels enhances treatment guidance and prognosis prediction in heart failure patients [34].
- DXA, recently defined as the gold standard, is an easy-to-use method with minimal radiation exposure and a lower cost than CT or MRI. However, fluctuations in body water make this measurement not sufficiently reliable for patients suffering from HF.
- Muscle ultrasound has emerged as a promising technique for measuring muscle mass due to its low cost, availability, simplicity and correlation with MRI findings. It allows for the differentiation between edema in subcutaneous tissue and lean tissue. Standardization and cutoff values have recently been published by a DRECO study relating to a Spanish population [35]. However, the validated cutoff points are not yet well defined in the population with HF.

##### Etiologic Criteria

GLIM criteria encompass two etiologic criteria: reduced food intake or absorption and the presence of inflammatory burden. HF is characterized by low-grade inflammation, a common feature in the progression of chronic disorders, leading to disruptions in body composition, as described in the pathophysiology section [5,6,8].

#### 4.1.3. Other Nutritional Indirect Diagnostic Methods

Various biochemical parameters, such as ghrelin, leptin, adiponectin, myostatin, C-terminal agrin fragment (CAF), TNF-α, IL-1, IL-6, growth hormone (GH)/IGF-1 and testosterone, have been explored as potential biomarkers for assessing nutritional status in patients with heart failure [8]. However, these parameters are not currently feasible for routine clinical practice and are usually confined to research [8].

Other indirect methods for assessing the nutritional status of HF patients have been described, such as arterial stiffness, which is measured by assessing aortic pulse wave velocity. Greater arterial stiffness is associated with lower skeletal muscle mass in older adults and patients with cardiovascular disease [36], serving as a robust predictor of future cardiovascular events and all-cause mortality.

On the other hand, some studies suggest that serum vitamin D levels may serve as a diagnostic marker for sarcopenia. A cross-sectional study demonstrated that low 1,25-dihydroxyvitamin D and low 25-hydroxyvitamin D (25-OHD) are linked to lower muscle strength, while a prospective longitudinal study found that lower serum 25-OHD levels increase the risk of sarcopenia in older men and women [9,37].

### 4.2. Sarcopenia Assessment: European Working Group of Sarcopenia in Older People Algorithm (EWGSOP2)

A challenge in comparing the prevalence and severity of sarcopenia across different clinical populations, such as patients with HF, is the lack of a unified definition of sarcopenia and a standardized diagnostic method. Depending on the region where the study is conducted, either Asian or European criteria are followed, which, despite being similar, complicate comparisons [2,38].

**Figure 6:**
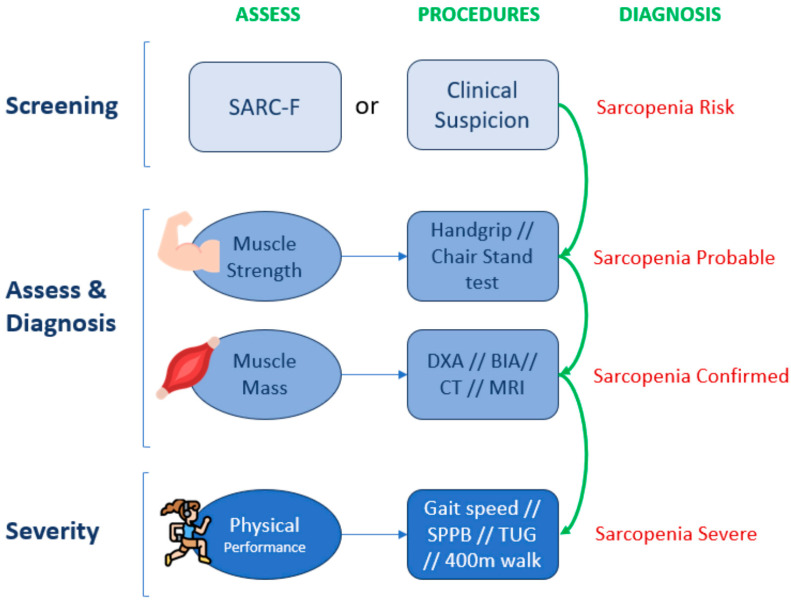
Sarcopenia diagnosis flowchart following EWGSOP2 algorithm for case-finding, making a diagnosis and quantifying severity in practice. Adapted from Cruz-Jentoff A. J. et al. 2019 [2]. SPPB: short physical performance battery; TUG: timed-up-and-go test.

In our setting, we adhere to the European Working Group criteria, which also published a revision in 2019, presenting a new diagnostic and severity algorithm for clinical practice (Figure 6).

#### 4.2.1. Screening

Currently, three validated questionnaires with different sensitivities and specificities are used to detect the risk of sarcopenia depending on the definition used (European or Asian groups): the Mini Sarcopenia Risk Assessment (MSRA) [39], which is available in two versions with five and seven questions, respectively (MSRA-5 and MSRA-7), and the Strength, Assistance with Walking, Rising from a Chair, Climbing Stairs, and Falls (SARC-F) questionnaire [40].

The European group recommends the use of the SARC-F questionnaire as the most appropriate screening method for sarcopenia in older adults, regardless of their underlying pathology.

#### 4.2.2. Diagnosis

If a positive screening result is obtained or if there is a suspicion that the muscle compartment may be affected, the first step is to assess the individual’s muscle strength.

If muscle strength is below the cutoff points for the technique used, it indicates a probable case of sarcopenia, where corrective measures should already be introduced to attempt to reverse the situation.

The recommended techniques include measuring the isometric strength of the muscles of the hand and forearm using a handgrip or assessing quadricep strength using a chair stand test [41,42]. Due to their simplicity, grip strength measurements are recommended for regular use in hospital practice, specialized clinical settings and community healthcare. Grip strength correlates with the strength of other body compartments, making it a reliable surrogate for more complex measurements of arm and leg strength. Therefore, in clinical practice, this is enough to trigger an assessment of underlying causes and start intervention [2].

To confirm the diagnosis of sarcopenia, it is necessary to demonstrate that muscle mass is reduced. This can be carried out through direct measurements using body composition techniques with validated cutoff points (DXA, BIA, CT and MRI), and the severity of sarcopenia would be determined in terms of functional impairment measured through the use of physical performance tests: SPPB (short physical performance battery), gait speed, TUG (timed-up-and-go test) and 400 m walk.

As a reminder, assessments of nutritional status and muscle mass are indispensable for all HF patients. While no specific or validated screening or diagnostic tests exist for heart failure, the GLIM criteria are valid for the diagnosis of malnutrition, and the EWGSOP2 algorithm is also valid for the diagnosis of sarcopenia in HF patients. Given the potential masking effect of edema on changes in patient weight/BMI, other methods of body assessment, such as bioelectrical vector impedance or muscle ultrasound, are advisable, particularly in patients with congestion. Biochemical parameters, such as hormones or several interleukins, are not currently applicable in routine clinical practice.

## 5. Therapeutic Approach

The early identification and treatment of malnutrition and sarcopenia are associated with improved outcomes in HF patients, including those requiring advanced therapies, such as ventricular assist devices (VADs) or heart transplantation. It should be noted that the specific recommendations for the nutritional assessment and treatment of patients with heart failure do not appear to have detailed guidance in current nutritional guidelines [37]. Consequently, adhering to general recommendations for malnutrition and sarcopenia management based on physical exercise and nutritional supplementation becomes imperative.

Key considerations in malnutrition management, such as oral health, which are closely tied to physical frailty and nutritional status in cardiovascular patients, warrant assessment. This is particularly crucial for elderly patients with multiple comorbidities [9,19,43].

### 5.1. Physical Exercise

Changes in the body composition of HF patients correlate with reduced exercise capacity, with a sedentary lifestyle being identified as a risk factor for sarcopenia, especially in symptomatic HF patients with a preserved ejection fraction [11]. Physical activity levels in HF patients are linked to mortality, health-related quality of life, major adverse cardiac events (MACEs) and length of hospital stay.

Recognizing physical activity as an indispensable complement to nutritional therapy [8] provides primary treatment options against the consequences of sarcopenia and cachexia in cases of HF [44].

Exercise training, an evidence-based therapeutic strategy, demonstrates prognostic benefits in cardiovascular diseases, including HF. It not only exhibits cardioprotective effects, retarding the progression from cardiac dysfunction to heart failure, but also triggers anticatabolic signaling in skeletal muscle, potentially through the induction of PGC1α [5].

#### Types of Exercise

Both aerobic and resistance exercise provide significant benefits to adults with and without HF, influencing body composition, glucose and lipid metabolism, cardiovascular dynamics and the reduction in the expression of pro-inflammatory cytokines, including myostatin [17,45,46]. Therefore, aerobic physical activity should be considered complementary nutritional therapy in HF patients.

Recommendations from the European Society of Cardiology (ESC) and American College of Cardiology/American Heart Association (ACC/AHA) emphasize regular aerobic exercise that results in mild or moderate respiratory distress, which is backed by robust evidence (level of evidence A, class I recommendation). Additionally, a systematic review showed that combining progressive resistance training with aerobic training in patients with coronary artery disease aged around 60 years enhances cardiopulmonary function and skeletal muscle strength compared to aerobic training alone. This suggests the viability of resistance training as a strategy to counteract skeletal muscle atrophy in subjects with HF and sarcopenia [43].

For HF patients with a preserved LVEF, an increase in absolute peak VO_2_ (the highest value of oxygen consumption obtained during a progressive ergometric exercise test) aligns with higher values of appendicular skeletal muscle mass. This supports the presumed role of muscle mass as being responsible for the reduced exercise tolerance observed in HFpEF patients, with improvement noted after exercise training [47].

Accelerometer-measured daily step count has been proposed as a prognostic indicator of mortality in HF patients. It is significantly lower in male cardiac patients with sarcopenia, emphasizing its utility in identifying elderly male patients with sarcopenia. The cutoff values reported were as follows: 3551.80 steps/day for 1 week; energy expenditure of physical activity of 85.17 kcal/day for 1 week [43].

Neuromuscular electrical stimulation in HF patients is emerging as an interesting therapeutic option, demonstrating safe improvements in functional capacity, muscle strength and quality of life compared to conventional aerobic exercise [17].

### 5.2. Nutrition

Malnutrition is a significant risk factor for poor outcomes in both general medical inpatients, specifically in patients with chronic heart failure. The clinical presentations of malnutrition vary, from appetite and weight loss to muscle mass reductions (sarcopenia) and severe cardiac cachexia. Hospitalized heart failure patients are at high risk of further nutritional deterioration. While guidelines recommend nutritional support, evidence specifically relating to patients with heart failure remains limited. During hospitalization and recovery, attention to dietary needs plays a crucial role in improving outcomes and overall wellbeing. Researchers have explored various aspects of this topic, emphasizing the impact of nutrition on patient health. Among these studies, certain findings stand out that emphasize the need for tailored nutritional interventions to effectively support heart failure patients. The EFFORT study has shown that malnutrition is a prognostic factor for both short- and long-term mortality in hospitalized heart failure patients. Individualized nutritional interventions, when compared to standard hospital nutrition, have been proven effective in reducing these risks [48]. In this way, a PICNIC study reported a >50% mortality reduction from any cause and a reduced risk of readmission due to worsening of HF over 6 months when receiving in-hospital nutritional support; however, potential harm exists relating to increased salt and fluid intake [49]. Furthermore, the NOURISH study reported a 50% mortality reduction in cardiopulmonary patients over 3 months in patients receiving in-hospital nutritional support compared with clinical practice [50]. However, due to insufficient trial data, no specific recommendations exist for nutritional support in hospitalized chronic heart failure patients.

Multidisciplinary approaches are recommended, emphasizing weight monitoring and avoiding excessive fluid/salt intake.

#### 5.2.1. Diet

There is a need for large-scale trials to explore the effects of dietary interventions on muscle mass and strength in individuals with heart failure.

Patients with heart failure require a higher protein intake than the general population to maintain their muscle mass due to increased anabolic resistance and reduced muscle perfusion. A study involving 2282 predominantly male (83%) patients with heart failure, with an average age of 68 years, revealed that those who consumed less protein had lower BMIs, more severe congestive symptoms and a higher mortality rate compared to those in the highest quartile of protein intake [51].

However, most societies support the adoption of a Mediterranean diet or a DASH (Dietary Approaches to Stop Hypertension) diet [4,5]:

- Most societies support the adoption of a Mediterranean diet (MedDiet) or dietary approaches such as the DASH diet by people with or at risk of developing HF.
- A Mediterranean diet has been shown to protect against the development of cardiovascular diseases despite the fact that there are currently conflicting results regarding its ability to reduce the risk of HF. Recent studies indicate that greater compliance with this diet can lead to a decrease in hospital readmissions due to this cause.
- On the other hand, regarding the DASH diet, small clinical trials focusing on patients with hypertensive HF with a preserved ejection fraction have shown a reduction in blood pressure, arterial stiffness and oxidative stress and improved left ventricular diastolic function. The DASH diet has also been associated with fewer readmissions after hospitalization due to HF and lower mortality.

Regarding sodium intake, the European Society of Cardiology (ESC) advises against excessive salt intake (>5 g/day) in HF patients [1]. However, the supporting evidence for this recommendation is limited, leading to divergent conclusions by different professional societies.

#### 5.2.2. Nutritional Supplements

In cases of insufficient oral intake, oral nutritional supplementation is advised, regardless of the presence of cardiac cachexia. High-calorie and high-protein supplements are recommended, with cautious consideration of caloric intake reductions advised for obese patients.

- Hypercaloric supplements (1.52 kcal/mL) and hyperproteic supplements are generally advised, potentially improving inflammation status, quality of life and survival in HF patients. A high protein goal of 1.1 g/kg or more could be necessary, especially when considering the results of a study focusing on the nitrogen balance of 57 non-obese patients with HF versus 49 controls [5]. Although there is no scientific literature to support screening for and treatment of cardiac cachexia, the strong association between cachexia and mortality suggests that it is reasonable to try to adapt the inadequate intake of proteins and calories in selected patients and that functional status may be improved when supplying a protein intake that exceeds the 0.8 g/kg/day value recommended for the general population [3,24,37]. A protein intake goal of 1.1 g/kg daily is reasonable (as per PROT-AGE and Academy of Nutrition and Dietetics recommendations) [4].
- Hypercaloric and hyperproteic oral nutritional supplementation (ONS) enriched with β-Hydroxy-β-MethylButyrate (HMB) demonstrated significant improvement in terms of quality of life, handgrip strength and nutritional status and also reduced mortality risk in patients hospitalized for cardiovascular and pulmonary events, such as congestive HF [50,52,53].
- ESC guidelines propose omega-3 polyunsaturated fatty acid (PUFA) supplementation, considering its potential benefits in reducing hospitalization due to cardiovascular causes and death in HF patients, with level-B evidence in terms of recommendation: class IIb [1].
- Branched-chain amino acid (BCAA) preparations, which are essential for skeletal muscle formation, may be beneficial in HF, promoting postoperative wound healing and recovery from muscle fatigue after exercise, as well as improving muscle strength. The pros and cons of BCAA supplementation may vary depending on the patient and their specific conditions. BCAA supplementation for patients with cardiac dysfunction, in whom metabolic dysfunction could easily be presumed, should be carefully considered [4].

There are no specific recommendations on enteral/parenteral nutrition administration focusing on HF patients in the current nutritional guidelines. However, when oral nutritional support is insufficient, supplementation through an enteral route is considered if the gastrointestinal tract is intact and available. Consideration must be given to the potential adverse effects of electrolyte imbalances, fluid retention and sodium retention associated with refeeding syndrome, which can precipitate congestive HF or worsen clinical manifestations in HF patients. Consequently, initiating feeding with a low kcal/kg ratio is essential. Vigilant monitoring and correction of electrolyte deficiencies, along with the administration of thiamine, should be prioritized. Where necessary, the judicious use of loop diuretics may be considered as part of the comprehensive management strategy.

Finally, it is imperative to recognize that sarcopenia may manifest in obese patients, warranting nutritional interventions to support optimal muscle health independent of their body mass index (BMI). Prioritizing protein intake over caloric consumption is essential in this cohort. Furthermore, the selection of supplements, particularly those enriched with HMB, known for their ability to promote muscle development without concurrent adipose tissue accumulation, holds particular significance [4,53].

#### 5.2.3. Micronutrients

Interventional studies exploring the supplementation of micronutrients, inclusive of vitamins and trace elements, have demonstrated a potential role in HF patients, although existing clinical practice guidelines offer no universal recommendations. The guidance advocates replacing detectable deficiencies.

Despite a varied geographic distribution among HF cohorts, insufficient micronutrient intake, encompassing calcium, magnesium, zinc, iron, thiamine, vitamins D, E, K and folate, has been noted. However, evidence substantiating clinically relevant deficiencies resulting from these inadequacies is limited as present [4]. Notably, vitamin D supplementation exhibited increased muscle strength, particularly in elderly individuals with serum 25(OH)D concentrations < 30 ng/mL compared to those exceeding 30 ng/mL.

In a small randomized clinical trial, 31 patients with heart failure with a reduced ejection fraction were randomly assigned to receive 1-alanyl-1-glutamine (8 g/d) plus a polyunsaturated fatty acid (6.5 g/d) versus placebo for 3 months [1]. Fat-free mass increased (from 54.4 ± 3.2 to 56.1 ± 2.5 kg; *p* = 0.04), but skeletal muscle function and potential markers remained unchanged [4].

Three supplementation strategies have achieved positive findings in randomized clinical trials and warrant special attention: thiamine, coenzyme Q10 (CoQ10) and iron [4]. Several small observational and randomized studies have suggested an association between thiamine repletion and improved LVEF, although higher-quality evidence would be needed to make any clinical recommendations [4]. Coenzyme Q10 doses ranging from 60 to 300 mg/d have been studied in HF, and some small trials have suggested that there are improvements in NYHA functional class, LVEF, exercise capacity, QoL and even mortality; however, others are neutral [4].

Iron deficiency occurs in up to 55% of patients with chronic heart failure (CHF) and in up to 80% of patients with acute HF (AHF) and can be present independently of anemia. The specific cause of iron deficiency in patients with heart failure remains unknown, although it may be caused by increased loss, reduced intake, absorption (i.e., malnutrition, intestinal congestion) and/or altered iron metabolism caused by the chronic inflammatory activation of heart failure [54]. To improve symptoms, exercise capacity and quality of life in patients with HF and LVEF < 45, it would be advisable to consider iron supplementation with intravenous ferric carboxymaltose. It should also be considered for decreasing rehospitalizations for HF in those patients with LVEF < 50% recently hospitalized due to worsening HF [1,54]. There are several ongoing trials whose results may provide further evidence of the effects of ferric carboxymaltose in patients with HFpEF [1].

Overall, there is limited data to support robust recommendations on micronutrient supplementation for patients with HF, as research in this area has predominantly involved small studies rather than large randomized trials. Additional empirical research is required to adequately guide future clinical practice [4]. It is important to remember that micronutrient supplementation will be carried out to address detected deficiencies, rather than systematically for all patients. Table 3 outlines potential micronutrient supplements for patients with HF, along with their potential benefits and risks, for assessment by the physician responsible.

### 5.3. Drugs

There are also potential therapeutic options regarding nutritional state and muscle wasting that have been tested in patients with HF and have shown beneficial clinical effects in relation to cachexia and sarcopenia, such as beta-blockers, angiotensin-converting-enzyme (ACE) inhibitors or angiotensin II receptor blockers (ARBs). However, no definitive treatment options are currently established. The main guidelines also specify that none of the therapies mentioned above appear to be proven and that their safety is unknown. In addition, studies focused specifically on cardiac cachexia are lacking [4].

Renin–angiotensin–aldosterone system inhibitors could play a role in the prevention and treatment of CVD-related sarcopenia. Improved endothelial function due to ACE inhibitors may contribute to increased muscle blood flow and improve muscle function. ACE inhibitors and angiotensin II receptor blockers were reported to have muscle-protective properties [17,62].

The mineralocorticoid receptor antagonist spironolactone improved forearm endothelial function due to increased NO bioactivity despite chronic ACE inhibition. Thus, spironolactone may possibly delay the progression of sarcopenia by reducing skeletal myocyte apoptosis, improving vascular endothelial function and enhancing muscle contractility [63].

Finally, there is a possibility that treatment with sodium–glucose cotransporter 2 (SGLT2) inhibitors does result in a loss of skeletal muscle mass, even if the decrease is small. When SGLT2 inhibitors are used, combined therapy with exercise may be necessary to prevent skeletal muscle atrophy [10,64].

As a reminder, a combination of both aerobic and resistance exercise and nutritional supplementation should be included in the therapeutic approach used for patients with HF and muscle wasting. A high-calorie and high-protein diet should be encouraged for these patients while loosening restrictions on salt intake to improve palatability and intake. Patients with this profile may benefit from specific supplementation with HMB or PUFA as well as supplementation with micronutrients, such as coenzyme Q10. The emerging use of SGLT2 inhibitors should be monitored in the long term, given their potential catabolic effect on muscles.

## 6. Conclusions

The association between cardiac cachexia and sarcopenia in heart failure (HF) patients underscores their impact on disease severity and prognostic outcomes. Despite challenges in diagnostic methodologies, prioritizing nutritional screening and proactive prevention of sarcopenia is crucial. The Global Leadership Initiative on Malnutrition (GLIM) guidelines provide valuable insights. Efforts to establish well-defined protocols, including optimal dosages and durations of therapeutic interventions, are essential. Future research should focus on effective nutritional screening tools and interventions for HF patients with sarcopenia, sarcopenic obesity and cachexia.

## Figures and Tables

**Table 1:** Known skeletal muscle-secreted myokine and heart failure-related actions. Table based and modified from contents of the open-access articles published by Barbalho S.M [14] and by Berezin A.E [16].

Name of Myokine	Origin of Myokines	HF-Related Actions
Decorin	Skeletal Muscles	Downregulated in HF
	Fibroblasts	↓ Cardiac hypertrophy
	Vascular endothelial cells	↑ Cardiac fibrosis
	Cardiac myocytes	
	Smooth muscle cells	
Irisin	Skeletal Muscles	Downregulated in HF
	Myocardium	↓ Tolerance for physical activity
		↑ Skeletal muscle hypotrophy
Myonectin	Skeletal Muscles	Downregulated in HF
	Adipose tissue	↑ Skeletal muscle hypotrophy
Brain Derived Neurotropic Factor (BDNF)	Skeletal Muscles	Downregulated in HF
	Cardiac myocytes	↑ Tolerance for physical activity
	smooth muscle cells	
	Endothelial cells	
	Astrocytes	
Growth Differential Factor-11 (GDF-11)	Skeletal Muscles	Downregulated in HF
	Neural stem cells	↓ Physical endurance
	Cardiac myocytes	↑ Muscle weakness
		↑ Skeletal muscle hypotrophy
Myostatin	Skeletal Muscles	Upregulated in HF
	Cardiac myocytes	↑ Skeletal muscle hypotrophy
		↓ Tolerance for physical activity
		↑ Insulin resistance
		↑ Autophagy
		↑ Muscle weakness
Osteonectin	Skeletal Muscles	Upregulated in HF
	Adipose tissue	Predictor of poor HF outcomes
	Cardiac myocytes	↑ Cardiac contractility
	Bones mucosa	↑ Cardiac reparation at early stage
	Vasculature	↓ Vascular integrity at late stage
	Kidney and liver	↓ Cardiac myocyte survival

↑: increase. ↓: decrease.

**Table 2:** Known non-skeletal muscle-secreted myokine and heart failure-related actions. Table based and modified from contents of the open-access articles published by Barbalho S.M [14] and by Berezin A.E [16].

Name of Myokine	Origin of Myokines	HF-Related Actions
IL-8	Mononuclear phagocytes	Upregulated in HF
	Adipocytes	↓ Skeletal muscle energy metabolism
	Epithelial cells	↑ Cardiac fibrosis
	Endothelial cells	
	Mesenchymal Cells	
FGF-21	Cardiac myocytes	Downregulated in HF
	Pancreas	↑ Skeletal muscle mass
	Adipose tissue	↑ Tolerance for physical activity
	Liver	↓ Insulin Resistance
	Brain	
	Kidney	
IL-15	Cardiac myocytes	Downregulated in HF
	Mononuclear phagocytes	↑ Tolerance for physical activity
		↑ Skeletal muscle mass
		↓ White adipose tissue
		↓ Apoptosis of cardiac myocytes and myoblasts

↑: increase. ↓: decrease.

**Table 3:** Potential micronutrient supplementation in HF: benefits and risks [1,4,54,55,56,57,58,59,60,61].

Micronutrient	Benefits	Potentially Dangerous Side Effects (in General Adult Population)
Vitamin D	Improves muscle strength in elderly individuals with serum 25(OH)D concentrations < 30 ng/mL compared to those exceeding 30 ng/mL	Doses > 250 µg/d may produce signs of hypercalcemia, hypercalciuria and soft-tissue calcification.
Vitamin E	Possible improvement in markers of oxidative stress.	High doses of supplementation increase the risk of developing HF in patients after heart stroke. Increase the risk of prostate cancer. Rarely elevate creatine levels in urine. Nausea, diarrhea, intestinal cramps, fatigue, weakness, headache, blurred vision, rash, and gonadal dysfunction.
Vitamin B1 (thiamine)	Possible improvement in ejection fraction.	Not described
Vitamin B12	Improves respiratory muscle strength. Consider supplementation for patients on diuretics and restrictive diets.	Not described
Vitamin C	Improves contractility and endothelial function.	Very large doses (g quantity) may produce oxalate kidney stones in males.
Coenzyme Q10	Reduction in adverse cardiovascular events (mortality, hospitalizations, dyspnea, fatigue, arrhythmias, acute lung edema, nocturia, etc.). Improvement in NYHA functional class, improvement in the 6 min walk test, increase in exercise time.	Not described
Iron	Intravenous ferric carboxymaltose improves functional class, exercise capacity, and quality of life (These results are not observed when oral iron is given).	Ingestions ≥ 60 mg/kg of elemental iron are associated with serious toxicity and death.
Selenium	Scarce evidence. Seems to reduce serum insulin levels and insulin resistance (measured using the HOMA homeostasis model assessment of insulin resistance), as well as to improve the lipid profile.	Megadoses (>400 µg/d) could produce peripheral neuropathy, hair loss, nail changes, emesis, nausea, diarrhea, mental status changes, and visual loss.
